# A multiplex pedigree with pathologically confirmed multiple system atrophy and Parkinson’s disease with dementia

**DOI:** 10.1093/braincomms/fcac175

**Published:** 2022-07-04

**Authors:** Alessandra Fanciulli, Fabian Leys, Fabienne Lehner, Victoria Sidoroff, Viktoria C Ruf, Cecilia Raccagni, Philipp Mahlknecht, Demy J S Kuipers, Wilfred F J van IJcken, Heike Stockner, Thomas Musacchio, Jens Volkmann, Camelia Maria Monoranu, Iva Stankovic, Guido Breedveld, Federico Ferraro, Christina Fevga, Otto Windl, Jochen Herms, Stefan Kiechl, Werner Poewe, Klaus Seppi, Nadia Stefanova, Sonja W Scholz, Vincenzo Bonifati, Gregor K Wenning

**Affiliations:** Department of Neurology, Medical University of Innsbruck, Innsbruck, Austria; Department of Neurology, Medical University of Innsbruck, Innsbruck, Austria; Department of Neurology, Medical University of Innsbruck, Innsbruck, Austria; Department of Neurology, Medical University of Innsbruck, Innsbruck, Austria; Center for Neuropathology and Prion Research, Ludwig-Maximilians-University Munich, Munich, Germany; Department of Neurology, Medical University of Innsbruck, Innsbruck, Austria; Department of Neurology, Regional General Hospital Bolzano, Bolzano, Italy; Department of Neurology, Medical University of Innsbruck, Innsbruck, Austria; Department of Clinical Genetics, Erasmus MC, University Medical Center, Rotterdam, the Netherlands; Center for Biomics, Erasmus MC, University Medical Center, Rotterdam, the Netherlands; Department of Neurology, Medical University of Innsbruck, Innsbruck, Austria; Department of Neurology, University of Würzburg, Würzburg, Germany; Department of Neurology, University of Würzburg, Würzburg, Germany; Department of Neuropathology, Institute of Pathology, University of Würzburg, Würzburg, Germany; Neurology Clinic, Clinical Center of Serbia, University of Belgrade, Belgrade, Serbia; Department of Clinical Genetics, Erasmus MC, University Medical Center, Rotterdam, the Netherlands; Department of Clinical Genetics, Erasmus MC, University Medical Center, Rotterdam, the Netherlands; Department of Clinical Genetics, Erasmus MC, University Medical Center, Rotterdam, the Netherlands; Center for Neuropathology and Prion Research, Ludwig-Maximilians-University Munich, Munich, Germany; Center for Neuropathology and Prion Research, Ludwig-Maximilians-University Munich, Munich, Germany; Department of Neurology, Medical University of Innsbruck, Innsbruck, Austria; Department of Neurology, Medical University of Innsbruck, Innsbruck, Austria; Department of Neurology, Medical University of Innsbruck, Innsbruck, Austria; Department of Neurology, Medical University of Innsbruck, Innsbruck, Austria; Neurodegenerative Diseases Research Unit, National Institute of Neurological Disorders and Stroke, Bethesda, MD, USA; Department of Neurology, Johns Hopkins University Medical Center, Baltimore, MD, USA; Department of Clinical Genetics, Erasmus MC, University Medical Center, Rotterdam, the Netherlands; Department of Neurology, Medical University of Innsbruck, Innsbruck, Austria

**Keywords:** multiple system atrophy, Parkinson’s disease, Parkinson’s disease with dementia, multiplex pedigree, genetics

## Abstract

Multiple system atrophy is considered a sporadic disease, but neuropathologically confirmed cases with a family history of parkinsonism have been occasionally described. Here we report a North-Bavarian (colloquially, Lion’s tail region) six-generation pedigree, including neuropathologically confirmed multiple system atrophy and Parkinson’s disease with dementia.

Between 2012 and 2020, we examined all living and consenting family members of age and calculated the risk of prodromal Parkinson’s disease in those without overt parkinsonism. The index case and one paternal cousin with Parkinson’s disease with dementia died at follow-up and underwent neuropathological examination. Genetic analysis was performed in both and another family member with Parkinson’s disease. The index case was a female patient with cerebellar variant multiple system atrophy and a positive maternal and paternal family history for Parkinson’s disease and dementia in multiple generations. The families of the index case and her spouse were genealogically related, and one of the spouse's siblings met the criteria for possible prodromal Parkinson’s disease. Neuropathological examination confirmed multiple system atrophy in the index case and advanced Lewy body disease, as well as tau pathology in her cousin. A comprehensive analysis of genes known to cause hereditary forms of parkinsonism or multiple system atrophy lookalikes was unremarkable in the index case and the other two affected family members. Here, we report an extensive European pedigree with multiple system atrophy and Parkinson`s disease suggesting a complex underlying α-synucleinopathy as confirmed on neuropathological examination. The exclusion of known genetic causes of parkinsonism or multiple system atrophy lookalikes suggests that variants in additional, still unknown genes, linked to α-synucleinopathy lesions underlie such neurodegenerative clustering.

## Teaser

Fanciulli *et al*. report a Bavarian multiplex pedigree, including definite multiple system atrophy and Parkinson’s disease with dementia, in which no known causes of hereditary parkinsonism or multiple system atrophy lookalikes were found at in-depth genetic analysis. Genetic, still unknown, variants linked to α-synucleinopathy lesions may underlie such neurodegenerative clustering.

## Graphical Abstract

**Graphical Abstract:**
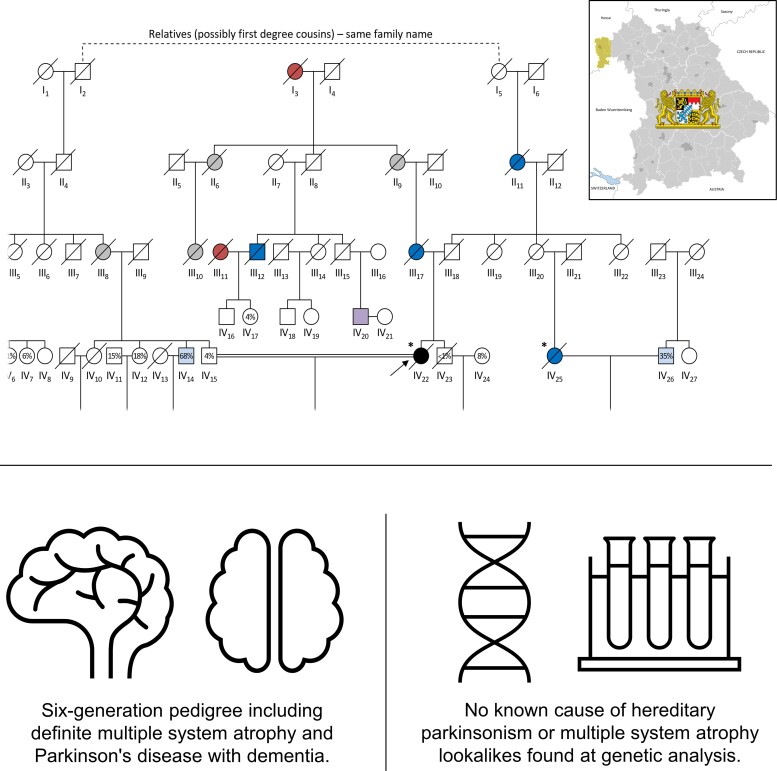

## Introduction

Multiple system atrophy is a rare, adult-onset, fatal neurodegenerative disease presenting with severe autonomic failure, poorly L-Dopa responsive parkinsonism, cerebellar and pyramidal features in various combinations.^1^ It is classified into multiple system atrophy-Parkinson, if parkinsonism prevails, or multiple system atrophy-cerebellar, if cerebellar ataxia predominates.

Widespread oligodendroglial cytoplasmic inclusions (GCIs or *Papp-Lantos bodies*) associated with striatonigral or olivopontocerebellar neurodegeneration are the histological hallmark of multiple system atrophy.^2^ The main constituent of the GCIs is misfolded α-synuclein,^3^ which classifies multiple system atrophy as oligodendroglial α-synucleinopathy, while neuronal α-synuclein aggregates (*Lewy bodies*) characterize Parkinson’s disease^4^ and dementia with Lewy bodies.^5^

Several longitudinal studies reported on environmental and behavioural factors, which increase the risk of developing Parkinson’s disease later in life.^6^ Genetic factors also contribute to the pathogenesis of Parkinson’s disease, with both established monogenic forms of the disease^4^ and variants in multiple genetic loci, which have been consistently associated with an increased risk of Parkinson’s disease.^7^

By contrast, no clear environmental or genetic risk factors have been found for multiple system atrophy, which is therefore considered a sporadic disease.^1,8^ Nevertheless, up to 18% of multiple system atrophy patients may have first-degree relatives affected by parkinsonism,^9–14^ and both European and Asian pedigrees of neuropathologically confirmed multiple system atrophy with an autosomal-dominant or recessive inheritance pattern have been reported.^15–18^

**Figure 1:**
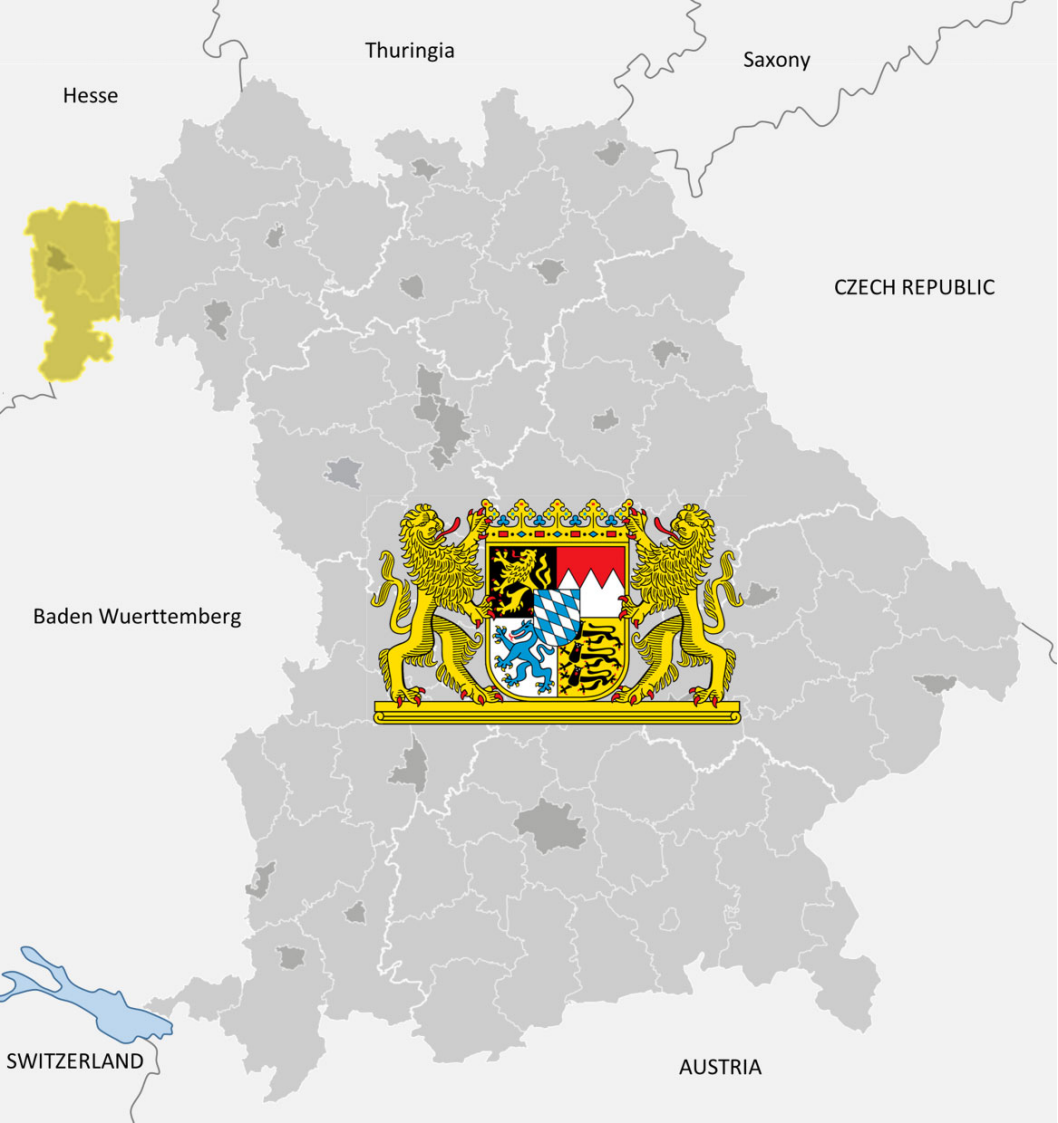
**Map of Bavaria indicating the pedigree’s origin.** Created with Microsoft PowerPoint 2016 using files from Wikimedia Commons that are available under the Creative Commons CC0 1.0 Universal Public Domain Dedication (Bavaria Blank Map on Wikimedia Commons. Accessed 02 February 2022. https://commons.wikimedia.org/wiki/File:Bayern_Blank_map.svg) and from the Bavarian Government, which has released its Coat of Arms into the public domain (Coat of Arms of Bavaria on Wikimedia Commons. Accessed 02 February 2022. https://commons.wikimedia.org/wiki/File:Coat_of_arms_of_Bavaria.svg).

Here we report the clinical, genetic, and neuropathological characteristics of a six-generation multiplex pedigree, including neuropathologically confirmed multiple system atrophy (index case) and Parkinson’s disease with dementia. The pedigree originated from the Bavarian Lower Main, a region in the far northwest of Bavaria colloquially known as the ‘lion’s tail’ since the Bavarian lion became the heraldic animal of the house of Wittelsbach in 1214 (Fig. 1).^19^

## Material and methods

### Clinical characterization of the pedigree

The index case was a female patient with multiple system atrophy of cerebellar type treated at the Department of Neurology of the Medical University of Innsbruck, Austria. Prompted by her positive family history, between 2012 and 2020, we studied all living and consenting family members of age with a structured interview and clinical examination. This included the following:

- general demographic information, education, occupation, smoking habit, professional exposure to organic solvents, plastic monomers, pesticides or metal dusts, comorbidities and medication schedule;
- questionnaires for parkinsonian non-motor symptoms (Scale for Outcomes in Parkinson’s disease—Autonomic domain,^20^ Non-Motor Symptoms Scale,^21^ Schrag Quality of Life Questionnaire,^22^ Orthostatic Hypotension Questionnaire,^23^ REM Sleep Behavior Disorder—1 Question,^24^ Innsbruck REM Sleep Behavior Disorder Inventory,^25^ STOP BANG sleep apnoea screening,^26^ Montreal Cognitive Assessment;^27^
- detailed physical and neurological examination;
- scales for parkinsonism (Movement Disorder Society—Unified Parkinson’s disease rating scale,^28^ Unified Multiple System Atrophy Rating Scale,^29^ Hoehn & Yahr stage);^30^
- olfactory screening with the 16-items Sniffin’ sticks identification test (Burghart Messtechnik—Wedel, Germany);^31^
- measurement of supine to standing heart rate and blood pressure changes to screen for neurogenic orthostatic hypotension.^32–34^

In living family members with overt parkinsonism, we collected structured information on the disease and medication history. We checked the given neurological diagnoses against the current diagnostic criteria for multiple system atrophy,^8^ Parkinson’s disease,^35^ and Parkinson’s disease with dementia,^36^ as well as expert recommendations for drug-induced parkinsonism.^37^

### Family history

We interviewed all recruited family members for the presence of consanguinity, neurological or other diseases in previous generations. We asked each member, if they ever observed or were told that their ancestors had suffered from cognitive impairment, slowness of movements, tremor (if present, whether at rest or while handling objects, and which was the most affected part of the body), gait impairment, dream enacting behaviour, syncope, orthostatic dizziness or bladder disturbances. We cross-checked statements on the same ancestors among different family members.

### Calculation of the prodromal Parkinson’s disease risk in family members without overt parkinsonism

In the examined family members who did not show clinically overt parkinsonism at last available visit,^38^ we calculated the probability of suffering from prodromal Parkinson’s disease by applying the updated Movement Disorder Society research criteria for prodromal Parkinson’s disease.^39,40^ This is an evidence-based conceptual framework, which enables a probability diagnosis of prodromal Parkinson’s disease at a stage wherein early signs of Parkinson’s disease are already present, but do not yet fulfil the diagnostic criteria for overt Parkinson’s disease.^35^ A detailed description of the criteria for prodromal Parkinson’s disease is provided elsewhere.^39,40^ Briefly, the pre-test probability of prodromal Parkinson’s disease is based on age (i.e. estimated age-adjusted prevalence of prodromal Parkinson’s disease) and determines a minimum required likelihood ratio (LR) threshold, which must be exceeded for a diagnosis of probable prodromal Parkinson’s disease.^39^ LRs of available test positive (LR+) and test negative (LR−) risk factors, as well as prodromal markers are multiplied together to generate a total LR of prodromal Parkinson’s disease for that given person.^39^ If the total LR exceeds the pre-test age-adjusted minimum LR (i.e. probability threshold of 80% certainty), the diagnosis of probable prodromal Parkinson’s disease can be made, between 30 and 80% of possible prodromal Parkinson’s disease.^39,41^

For subjects aged 50 years or older, we based the pre-test probability and age-adjusted minimum LR on the recommended criteria.^39^ For subjects aged younger than 50 years of age, we used a pre-test probability of 0.2% and a minimum LR threshold of 1200, as applied in Mirelman *et al.*^42^

The individual cumulative LR was calculated on the Movement Disorder Society Web-portal for prodromal Parkinson’s disease Research (Accessed on 02 February 2022. www.movementdisorders.org/pdcalculator).^40^  Supplementary Table 1 provides an overview of the criteria for applying LR+, LR- or a neutral LR of 1 (LR1) for each prodromal Parkinson’s disease risk factor. Generally, all risk- and prodromal markers were defined as proposed in the updated Movement Disorder Society Research Criteria.^39,40^ We used a conservative approach by applying LR+ or LR− only if risk- and prodromal markers were unambiguously present or absent. For borderline cases or whenever unable to exclude possible differential diagnosis or confounding factors (e.g. medications), LR1 was applied.^39^ Missing values were also included in the calculation with LR1.

### Neuropathological examination

Both the index case (IV_22_) and the next of kin of the family member IV_25_, who deceased at follow-up to Parkinson’s disease with dementia, consented to donate their brains for research purposes to the Neurobiobank of the Ludwig-Maximilians-University in Munich, Germany. After removal, one brain hemisphere was stored at −80°C and the other one fixed in 4% buffered formalin. Tissue blocks from different brain regions including frontal, temporal, parietal and occipital neocortices, as well as basal ganglia, thalamus, amygdala, hippocampus, midbrain, pons, medulla and cerebellum were sampled from the formalin-fixed hemisphere and embedded in paraffin for histological examination. Paraffin-embedded sections (5 µm) were stained with routine methods including haematoxylin and eosin, Elastica van Gieson, Gallyas silver staining, Kluver-Barrera and Luxol fast blue/Periodic acid-Schiff. Additional immunohistochemistry and immunofluorescence analysis was performed using the primary antibodies listed in Table 1. For immunohistochemical staining, signals were detected using the iVIEW DAB Detection Kit or the ultraView (α-Synuclein, α-pTDP-43) Universal DAB Detection Kit (Ventana/Roche Diagnostics, Oro Valley, Arizona, USA). Immunohistochemical images were acquired on an Olympus BX41 brightfield microscope using the SC30 Color Camera and analyzed with the cellCens Standard software package (Olympus, Shinjuku, Japan). Immunofluorescence signals were detected with Alexa Fluor 488/546 anti mouse/rabbit antibodies (Invitrogen, Darmstadt, Germany) at a dilution of 1:300. Fluorescent images were recorded on a Zeiss Axioscan Z1 slide scanner and analyzed using the ZEN 3.1 (blue edition) software package (Zeiss, Oberkochen, Germany). The Gilman 2008 criteria were used to define multiple system atrophy-related neuropathological changes.^8^ The severity degree of Lewy body pathology was classified according to the Braak 2003 and Mc Keith 2005 criteria.^43,44^ For Alzheimer pathology the Braak and Braak 1991,^45^ Thal 2002,^46^ CERAD 2008 and NIA 2012 classification systems were applied.^47,48^ Argyrophilic grain disease was classified following the Saito 2004 criteria,^49^ microangiopathic changes and amyloid angiopathy according to the Thal 2002 and 2003 ones.^50,51^

**Table 1:** Primary antibodies used for the immunohistochemistry and immunofluorescence neuropathological examinations of IV_22_ and IV_25_^52^

Antibody against (clone)	Clonality	Supplier	Application	Dilution
α-Synuclein (clone 42)	Monoclonal	BD Biosciences	IHC/IF	1:2000 (IHC); 1:500 (IF)
α-Amyloid (4G8)	Monoclonal	Covance	IHC	1:5000
Phospho-Tau (AT8)	Polyclonal	Thermo Scientific	IHC	1:200
Cr3/43 (HLA-DP-DR-DQ)	Monoclonal	Agilent Technologies	IHC	1:100
FUS	Polyclonal	Sigma-Aldrich	IHC	1:100
GFAP	Polyclonal	Agilent Technologies	IHC/IF	1:2000 (IHC); 1:50 (IF)
Iba-1	Polyclonal	Wako	IF	1:500
Olig2	Monoclonal	Abcam	IF	1:25
p62 (Ick ligand; clone 3)	Monoclonal	BD Biosciences	IHC	1:100
pTDP-43 (Ser409/Ser410; Clone 1D3)	Monoclonal	own production^52^	IHC	1:50

IHC = immunohistochemistry; IF = immunofluorescence; FUS = fused in sarcoma protein; GFAP = glial fibrillary acidic protein; Iba-1 = ionized calcium-binding adapter molecule 1; Olig2 = oligodendrocyte transcription factor.

### Genetic analysis

Blood samples were collected from all living and consenting family members of age. Here we performed a genetic analysis of the index case (IV_22_) and her direct relatives with Parkinson’s disease (III_12_) and Parkinson’s disease with dementia (IV_25_).

Genomic DNA was isolated from peripheral blood lymphocytes using standard methods. Whole exome sequencing of the index case (IV_22_) and of the individual III_12_ was performed at the Center for Biomics of the Erasmus MC, the Netherlands, by using the SureSelect clinical relevant exomes capture kit (Agilent, Santa Clara, CA) and Illumina HiSeq4000, paired-end 150 base pairs sequencing (Illumina, San Diego, CA). We aligned the data to the human reference genome hg19/GRCh37 using the Burrows-Wheeler Aligner^53^ and called variants with the Genome Analysis Toolkit.^54^

Whole exome sequencing on the individual IV_25_ was performed later on by CENTOGENE GmbH (Rostock, Germany), using the Twist Human Core Exome Plus Kit (Twist Bioscience, San Francisco, CA) and the NovaSeq 6000 System (Illumina, San Diego, CA). The reads were processed according to the Genome Analysis Tool Kit 3.7 guidelines^54^ and aligned to the genome build GRCh37/hg19 with the Burrows-Wheeler Aligner.^53^ The variants were called with Genome Analysis Tool Kit HaplotypeCaller, freebayes (v1.1.0)^55^ and bcftools (v1.4.1)^56^ and annotated with the Whole Genome Sequencing Annotator v0.85.^57^

Whole exome sequencing data generated from both the index patient (IV_22_) and the family members IV_25_ and III_12_ were searched for variants in genes that

- may increase the risk for multiple system atrophy (*COQ2, MAPT, EDN1, ELOVL7, FBXO47, SHC2, TMEM230, ABI3, PLCG2*);^58^
- might cause diseases with multiple system atrophy-mimicking phenotypes (*ATXN1, ATXN2, ATXN3, CACNA1A, ATXN7, KLHL1, ATXN8OS, PPP2R2B, TBP, ATN1, NOP56, FMR1, FXN, DCTN1, SNCA, GBA, LRRK2, SPG7, SPG11, LMNB1, POLG, CYP27A1, PRNP, C9ORF72*);^59^
- might cause inherited forms of parkinsonism (*SNCA, LRRK2, VPS35, GBA, CHCHD2, PRKN, PARK7, PINK1, ATP13A2, PLA2G6, RAB39B, FBXO7, DNAJC6, SYNJ1, VPS13C, PTRHD1, LRP10*).^4,60^

Both heterozygous and homozygous variants were considered of interest if they were rare (minor allele frequency below 1% in the Genome Aggregation Database^61^ and in in-house data sets), had a coding effect (missense, frameshift, inframe insertion/deletion, startgain, startloss, stopgain, stoploss) and/or an effect on mRNA splicing predicted by at least one of four in-silico tools (Ada,^62^ RF,^62^ SpliceAI^63^ or SQUIRLS).^64^

The entire open reading frame of *GBA* was sequenced by Sanger methods in the index patient IV_22_ and family members IV_25_ and III_12_. To account for the presence of the highly similar pseudogene, *GBAP1*, we employed a previously described protocol for the specific amplification of *GBA*.^65^ We performed variant annotation as described for the variants identified by whole exome sequencing and searched for variants with a minor allele frequency below 5% and with a coding effect and/or a predicted effect on mRNA splicing by at least one out of four in-silico tools (Ada,^62^ RF,^62^ SpliceAI^63^ or SQUIRLS).^64^

Established Parkinson’s disease- or parkinsonism-causing genes were evaluated for copy number variations with Multiplex Ligation-dependent Probe Amplification assays (SALSA P051 and P052; MRC Holland, the Netherlands).

We additionally screened for DNA repeat expansions in the following genes: *ATXN2* (CAG expansion), *ATXN3* (CAG expansion), *CACNA1A* (CAG expansion), *TBP* (CAG expansion), *ATN1* (CAG expansion) and *RFC1* (AAGGG expansion) and assessed the *ApoE* status.

### Protocol approval, data management and informed consent

The Innsbruck ethical committee approved the clinical and genetic research protocol (Ethical committee No. AM1979d and 1274/2018), which were conducted in accordance with the Declaration of Helsinki and the European General Data Protection Regulation. All examined family members gave their written informed consent to participate in the study. The Munich Neurobiobank of the Ludwig-Maximilians-University received approval by the local ethical committee for collecting and storing brain tissue for research purposes and publishing the cases presented herewith in anonymized form (Ethical committee No. 345-13). The authors take full responsibility for the integrity of data.

### Data availability

Data not published within this article are available upon reasonable request for any qualified investigator.

## Results

### The clinical pedigree

**Figure 2:**
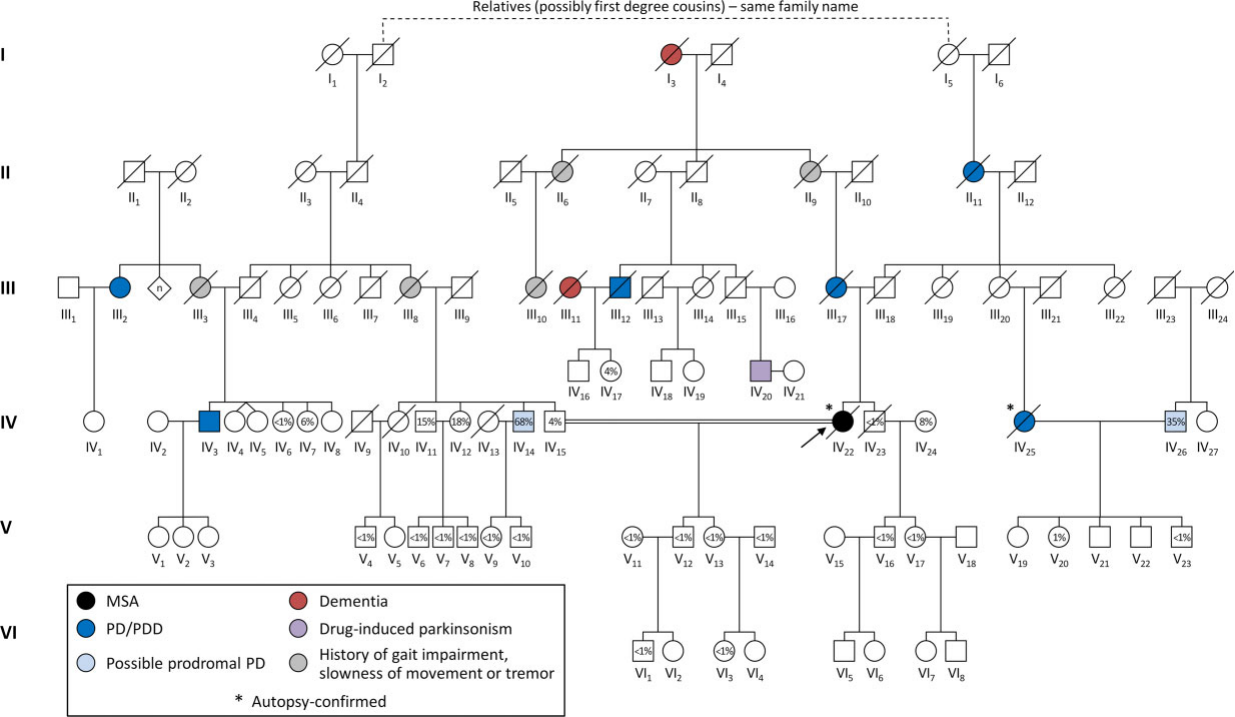
**Family tree of a Bavarian multiplex pedigree with pathologically confirmed multiple system atrophy and Parkinson’s disease with dementia.** The percentages within the symbols indicate the calculated probability of prodromal Parkinson’s disease in the examined family members without clinically evident parkinsonism. MSA = multiple system atrophy; PD = Parkinson’s disease; PDD = Parkinson’s disease with dementia.

In 2012, there were 59 living family members; the index case and three other family members (III_12_, IV_23_ and IV_25_) died during follow-up (Fig. 2). We collected information on generation I to III from interviews and available medical records, while we directly examined 30 members of generation IV to VI. Six members of generation VI were underage and therefore excluded from the study. Here we report the medical history of the family members with overt neurological features and diagnoses.

### Generation I

**Subject I_3_** suffered from severe dementia with psychotic episodes; she died at the age of 90.

**Subject I_2_ and I_5_** were related to each other, possibly first-degree cousins. It is unknown whether they suffered from any neurological disease.

### Generation II

**Subject II_11_** suffered from tremor-dominant Parkinson’s disease.

### Generation III

**Subject III_2_** is in her mid-90s and suffers from advanced Parkinson’s disease with dementia, which begun at the age of 70.

**Subject III_11_** suffered from Alzheimer’s disease beginning in her mid-60s, died in her 80s.

**Subject III_12_** was diagnosed with Parkinson’s disease at the age of 65. He suffered from severe constipation, olfactory dysfunction and voiding disturbances in his late years, but no cognitive impairment, REM sleep behaviour disorder or orthostatic intolerance. He died in his 90s to age-related causes.

**Subject III_17_** was diagnosed with Parkinson’s disease at the age of 75. She suffered from asymmetric rest and action tremor, short-stepped gait, falls, severe orthostatic intolerance, urinary incontinence and mild dementia; she died in her 80s.

### Generation IV

**Subject IV_3_** suffers from Parkinson’s disease since the age of 59. Twenty years into the disease, he is wheelchair-bound, demented, has visual hallucinations and urinary incontinence.

**Subject IV_20_** was diagnosed at the age of 62 with bipolar disorder, treated since then with different CNS-acting medications, including neuroleptics. At the age of 68, he showed mild left-sided parkinsonism without postural instability. ^123^I-Iofluopane SPECT showed bilaterally normal presynaptic dopaminergic imaging, indicating a drug-induced form of parkinsonism in this case.

**Subject IV_22_ (index case)** suffered from gait unsteadiness, falls, slurred speech, depression, urinary incontinence, orthostatic intolerance and dream enacting behaviour since the age of 55. Cerebral MRI showed olivopontocerebellar atrophy and polysomnography disclosed both REM sleep behaviour disorder and central sleep apnoea. In the following years, she developed overt cerebellar ataxia, cardiovascular and urological autonomic failure, fulfilling the criteria for probable multiple system atrophy of cerebellar type.^8^ She died at the age of 60 after 5 years of disease duration.

**Subject IV_25_** was diagnosed with Parkinson’s disease at the age of 55. She initially showed good L-Dopa responsiveness, but eventually developed disabling motor fluctuations, progressive gait and cognitive impairment. At the age of 78, she was demented, bed-ridden and showed speech apraxia, severe dysarthria, vertical and horizontal gaze palsy, positive frontal release signs, retrocollis and very severe left-sided parkinsonism. She died at the age of 79 to Parkinson’s disease with dementia.

### Prodromal Parkinson’s disease risk calculation

Twenty-six family members aged 18–85 years, which we examined between 2012 and 2020, did not fulfil the criteria for overt Parkinson’s disease or other movement disorders at last available follow-up and underwent a retrospective calculation of the prodromal Parkinson’s disease LR.

Supplementary Table 1 provides an overview of the available data and distribution thereof within the studied family members for each prodromal Parkinson’s disease risk marker. Intermediate strength genetic variants (*GBA* and *LRRK2* mutation), polygenic risk score, transcranial ultrasound, dopaminergic SPECT study, polysomnography, sensor-based motor testing and plasma urate were not studied and therefore rated with LR1.

None of the examined family members without clinically evident parkinsonism reached the threshold for probable prodromal Parkinson’s disease (see the individual prodromal Parkinson’s disease risk scores in Fig. 2). In generation IV, one sibling (IV_14_) of the spouse of the index case, whose families were related in the first generation, and one married-in member (IV_26_) reached the threshold of possible prodromal Parkinson’s disease.^39,41^

### Neuropathology

#### Subject IV_22_ (index case): definite multiple system atrophy

Macroscopic examination of the brain of the index case revealed severe atrophy of the brainstem and the cerebellum, widened ventricular system and bilateral paleness of the substantia nigra.

**Figure 3:**
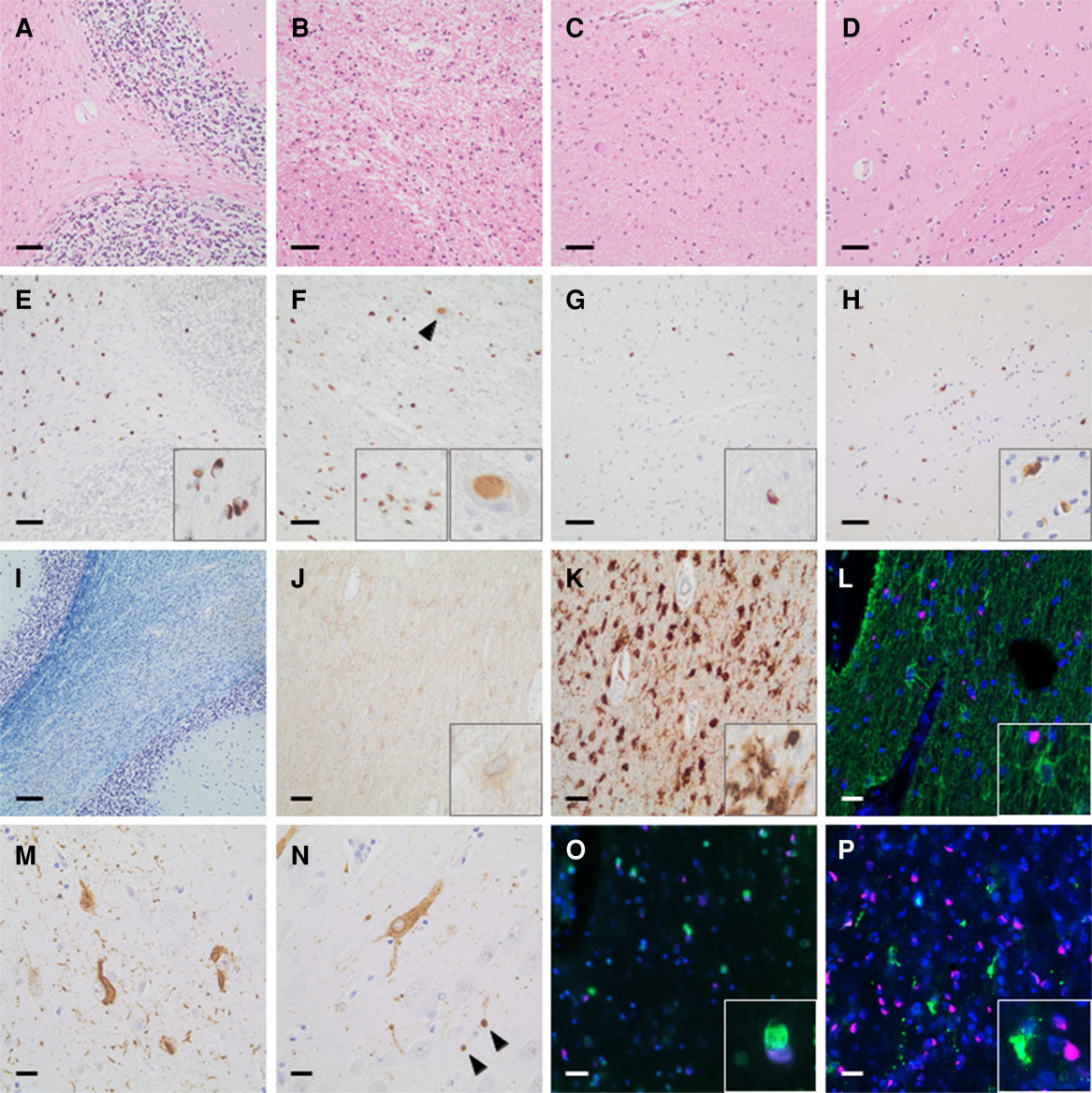
**Neuropathological examination of the index case IV_22_ with definite multiple system atrophy.** Substantial loss of Purkinje cells in the cerebellar cortex (**A**; haematoxylin & eosin) and marked demyelination (**I**; Kluver-Barrera), with reactive astro- (**J**; GFAP immunohistochemistry) and microgliosis (**K**; Iba-1 immunohistochemistry) of the cerebellar white matter. Severe neuronal loss, demyelination and reactive gliosis in the pons and inferior olive (**B–C**; haematoxylin & eosin), but not putamen (**D**; haematoxylin & eosin). High amounts of α-synuclein-positive glial cytoplasmic inclusions in the cerebellar white matter (**E**; α-synuclein immunohistochemistry) and pons (**F**; α-synuclein immunohistochemistry), with additional neuronal cytoplasmic inclusions there (**F**; arrowhead, inset; α-synuclein immunohistochemistry). Low to moderate amounts of glial cytoplasmic inclusions in the inferior olive (**G**; α-synuclein immunohistochemistry) and putamen (**H**; α-synuclein immunohistochemistry). α-synuclein inclusions in oligodendrocytes (**O**; α-synuclein/Olig2 double immunofluorescence), but not in astrocytes (**L**; α-synuclein/GFAP double immunofluorescence) or microglia cells (**P**; α-synuclein/Iba-1 double immunofluorescence) at double immunofluorescence. Early Alzheimer-associated changes with neurofibrillary tangles and neuropil threads in the (trans)-entorhinal cortex (**M**; AT8 immunohistochemistry). Early argyrophylic grain disease changes with pretangles and argyrophilic grains (arrowheads) in the hippocampus (CA1/2) (**N**; AT8 immunohistochemistry). Scale bars: A-H, 50 µm; I, 100 µm; J-P, 20 µm. GFAP = glial fibrillary acidic protein; Iba-1 = ionized calcium-binding adapter molecule 1; Olig2 = oligodendrocyte transcription factor.

In agreement with the cerebellar multiple system atrophy phenotype,^66^ the histological examination showed substantial loss of Purkinje cells in the cerebellar cortex (Fig. 3A) and marked demyelination of the cerebellar white matter (Fig. 3I) along with reactive astro- and microgliosis (Fig. 3J and K). Severe neuronal loss, reactive gliosis and demyelination were also observed in the pons and inferior olive, whereas the putamen was barely affected (Fig. 3B–D). We detected high amounts of GCIs in the cerebellar white matter and in the pons (Fig. 3E and F), and low to moderate amounts thereof in the inferior olive and the putamen (Fig. 3G, H). In addition, sparse neuronal cytoplasmic inclusions were observed in the pons (Fig. 3F, arrowhead, right inset). Double immunofluorescence staining confirmed α-synuclein inclusions in oligodendrocytes (Fig. 3O) but not in astrocytes or microglia cells (Fig. 3L and P). The main pathology was accompanied by early Alzheimer-associated changes with neurofibrillary tangles and neuropil threads in the (trans)-entorhinal cortex (Fig. 3M; Braak and Braak stage I; Thal phase 0; CERAD 0; NIA classification A1, B0, C0)^45–48^ early argyrophilic grain disease changes with pretangles and argyrophilic grains (arrowheads) in the hippocampus (CA1/2; Saito stage 1 Fig. 3N)^49^ and microangiopathic changes (Thal stage C).^51^ β-amyloid staining revealed Aβ depositions in leptomeningeal, as well as single intracerebral non-capillary vessels but no amyloid plaques (Thal stage 1, type 2).^50,51^ Staining for phosphorylated TDP-43 and fused in sarcoma protein gave negative results (data not shown).

#### Subject IV_25_: definite Lewy body disease

Macroscopic examination of the brain of the cousin of the index case, who died to Parkinson’s disease with dementia, showed moderate frontotemporal atrophy and ventricular widening, most pronounced in the occipital horn of the lateral ventricles.

**Figure 4:**
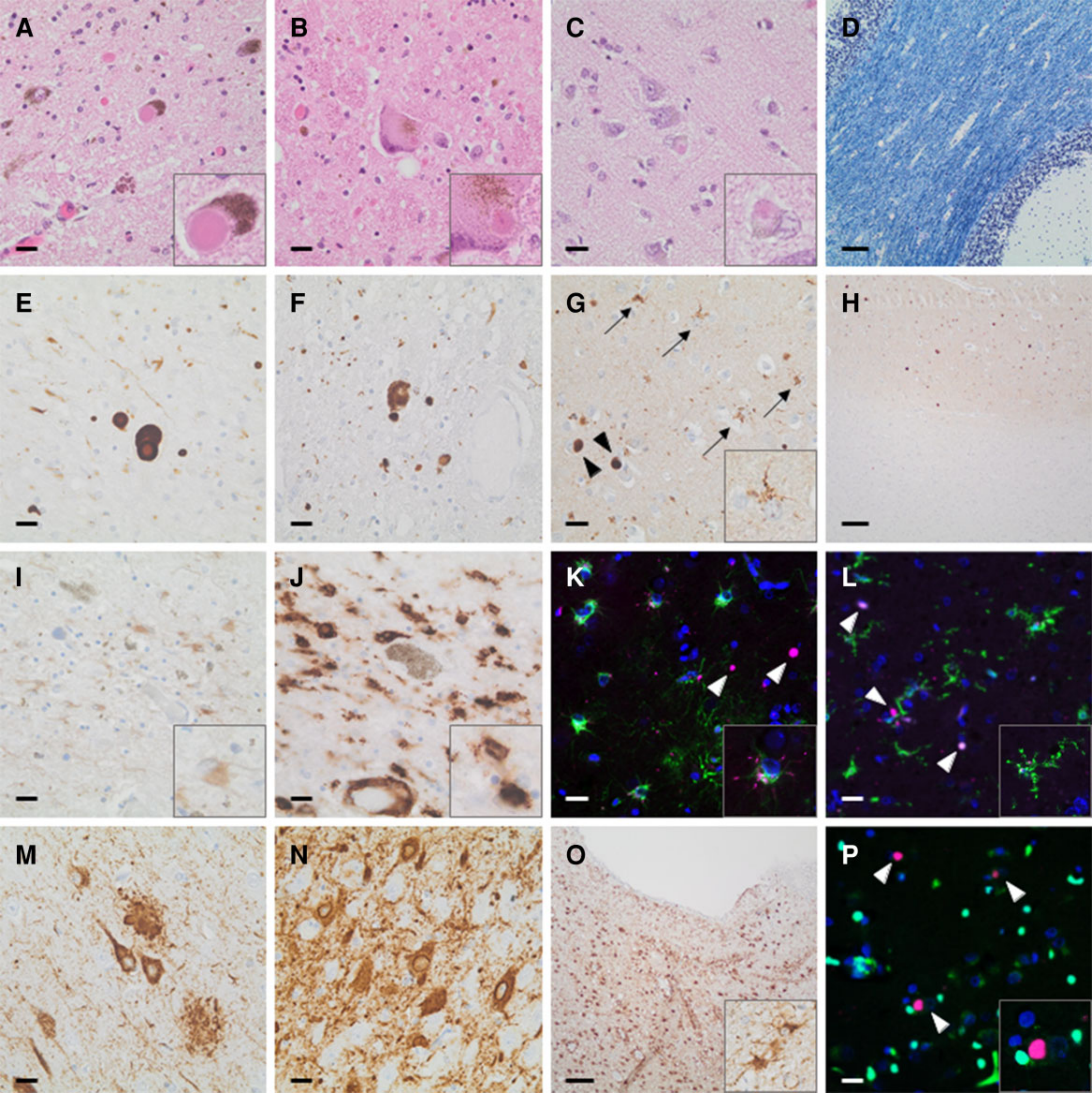
**Neuropathological examination of the family member IV_25_ deceased to Parkinson’s disease with dementia.** Lewy bodies and Lewy neurites strongly positive for α-synuclein detected in the substantia nigra (**A**, **E**; haematoxylin & eosin, α-synuclein immunohistochemistry), locus coeruleus (**B and F**; haematoxylin & eosin, α-synuclein immunohistochemistry) and neocortex (**C and G**; haematoxylin & eosin, α-synuclein immunohistochemistry), among other regions. Preserved myelination of the cerebral white matter (Kluver-Barrera, **D**). α-synuclein inclusions in numerous glial cells with fine, tiny processes (**G**; arrows, inset; α-synuclein immunohistochemistry) in the cortex, but not in the white matter (**H**; α-synuclein immunohistochemistry). α-synuclein-positive inclusions in astrocytes (**K**; α-synuclein/GFAP double immunofluorescence) and microglia cells (**L**; α-synuclein/Iba-1 double immunofluorescence), but not in oligodendrocytes (**P**; α-synuclein/Olig2 double immunofluorescence) next to Lewy bodies (arrowheads in K, L and P) at double immunofluorescence staining. Reactive astro- and microgliosis in the substantia nigra (**I and J**; GFAP and Iba-1 immunohistochemistry, respectively). Advanced Alzheimer’s disease pathology with neurofibrillary tangles, neuritic plaques, neuropil threads (**M**; AT8 immunohistochemistry). Advanced argyrophilic grain disease changes with pretangles and numerous argyrophilic grains (**N** arrowheads; AT8 immunohistochemistry). High amounts of thorn-shaped astrocytes at periventricular sites (**O**; AT8 immunohistochemistry). Scale bars: A–C, E–G, I, J, M, N: 20 µm; D, H, O: 100µm; L: 50 µm. GFAP = glial fibrillary acidic protein; Iba-1 = ionized calcium-binding adapter molecule 1; Olig2 = oligodendrocyte transcription factor.

On histological examination, we found severe neuronal loss and reactive gliosis (Fig. 4I and J) in the substantia nigra, IX and X cranial nerve nuclei and locus coeruleus. Lewy bodies and Lewy neurites were, among other regions, detected in the substantia nigra, locus coeruleus and neocortex on haematoxylin-eosin staining (Fig. 4A–C; Braak stage 6; diffuse neocortical McKeith subtype),^5,43,67^ as well as α-synuclein immunohistochemistry (Fig. 4E–H). In addition, we found α-synuclein-positive inclusions in numerous glial cells with fine, tiny processes in various cortical and subcortical regions (Fig. 4G, arrows and inset). However, no inclusions were present in the white matter (Fig. 4H). In contrast to the multiple system atrophy case, myelination of the white matter was largely preserved (Fig. 4D). Apart from neuronal Lewy bodies, double immunofluorescence staining revealed α-synuclein-positive inclusions in astrocytes and microglia cells (Fig. 4K and L), but not in oligodendrocytes (Fig. 4P). Middle-stage Alzheimer disease pathology with neuropil threads, neurofibrillary tangles and neuritic plaques (Fig. 4M; Braak and Braak stage IV; Thal phase 3; CERAD B; NIA classification A2, B2, C2)^45–48^, as well as advanced argyrophilic grain disease changes with pretangles and numerous argyrophilic grains (Fig. 4N, arrowheads; Saito stage 3)^49^ were also found. Moreover, we observed high amounts of thorn-shaped astrocytes at periventricular and perivascular sites, indicating aging-related tau-astrogliopathy (Fig. 4O), as well as cerebral and leptomeningeal microangiopathic changes (Thal stage C).^51^ Diffuse, as well as compact β-amyloid plaques were present in moderate to high density in the striatum, neocortical and temporomesial regions but no vascular Aβ deposition was found. TDP-43 and fused in sarcoma protein staining did not show any additional pathology.

#### Genetic analysis

In the cases IV_22_, III_12_ and IV_25_, whole exome sequencing achieved a mean read depth of 137×, 154×, and 105×, respectively. Within the genes of interest, the mean read depth was 150 × for IV_22_ with 94% of the regions above 30 × and 97% of the regions above 20x; 170 × for III_12_ with 95% of the regions above 30 × and 98% of the regions above 20×; and 97 × for IV_25_ with 98% of the regions above 30 × and 99% of the regions above 20×. The detailed distributions of the read depths in the genes of interest are provided in Supplementary Table 2. In IV_22_ and III_12_, we identified no variants of interest. In IV_25_, five variants were identified (Table 2), but considered of unknown significance. Two of these variants in the *ATXN7* gene, are present in the Genome Aggregation Database in >100 alleles and represent likely neutral polymorphisms. Screening of the *GBA* gene by a specific Sanger protocol revealed no variant of interest in the three family members analyzed. Multiplex ligation-dependent probe amplification assay detected no copy number variants in genes known to cause Parkinson’s disease or parkinsonism in any of the three family members. Pathogenic DNA repeat expansions were also not detected in any of the tested genes. The *ApoE* status was ɛ2/ɛ3 in the index multiple system atrophy case IV_22_, ɛ3/ɛ4 in her cousin with Parkinson’s disease with dementia (IV_25_) and ɛ3/ɛ3 in the other family member with Parkinson’s disease (III_12_).

**Table 2:** Variants of unknown significance identified in IV_25_^61,68^

Gene	*ATP13A2*	*ATXN7*	*ATXN7*	*CACNA1A*	*PLCG2*
Transcript	NM_022089	NM_000333	NM_000333	NM_023035	NM_002661
Coding DNA	c.2610-39G > T	c.118_119insAGCCGC	c.916A > T	c.3627_3629del	c.1343G > A
Protein	−	p.Gln39_Pro40insGlnPro	p.Ile306Phe	p.Glu1210del	p.Arg448Gln
Chromosome	1	3	3	19	16
Start Position (GRCh37)	17315008	63898390	63968025	13395957	81934366
Reference allele	C	.	A	TCC	G
Alternative allele	A	GCAGCC	T	.	A
Exon	Intron23	3	7	21	14
dbSNP	-	rs770364745	rs140270787	rs750826355	rs772575043
Zygosity	heterozygous	heterozygous	heterozygous	heterozygous	heterozygous
gnomAD v2.1.1	1/248252	106/57846	342/280872	15/278216	7/248484
CADD score	5.4	NA	24.8	NA	23.6
Predicted splicing effect	+	−	−	−	−

dbSNP = single nucleotide polymorphism database; gnomAD = Genome Aggregation Database;^61^ CADD = Combined Annotation Dependent Depletion;^68^  + = splicing effect predicted for the reported transcript; - = splicing effect not predicted for the reported transcript.

### Discussion

In case series of patients with neuropathologically confirmed multiple system atrophy, 13% had at least one first-, second- or third-degree relative with parkinsonism, whereas in other clinical series, the frequency rose to 18% among first-degree relatives.^11,14^ More recently, five pedigrees with pathologically confirmed multiple system atrophy and positive first-degree family history for Parkinson’s disease or multiple system atrophy were reported in the literature: three Japanese,^15,18,69^ one American^70^ and one German.^17^ In one of the Japanese pedigrees, in which the multiple system atrophy patients were siblings from a consanguineous marriage, a homozygous loss-of-function mutation in the *COQ2* gene, coding for the Coenzyme Q_10_ synthesizing enzyme, was later found. *COQ2* mutations have been found in other Japanese familial and sporadic multiple system atrophy cases, but not in North American and European multiple system atrophy natives.^71,72^ Similarly, a discordant loss of copy number of the *SHC2* gene was observed in monozygotic twins and sporadic Japanese multiple system atrophy patients but not in American ones.^73,74^ As mentioned before, α-synuclein is the main constituent of the GCIs, classifying multiple system atrophy as an oligodendroglial α-synucleinopathy.^3^ Even though an *A53T* mutation in the *SNCA* gene was found in a British family with autosomal-dominant early-onset Parkinson’s disease and both Lewy bodies and multiple system atrophy-like brain pathology,^75^ no *SNCA* dosage alterations, pathogenic point mutations or variants that may increase the risk of developing multiple system atrophy have been clearly identified.^58,76^ Mutations in the *LRRK2* gene, a common cause of monogenic Parkinson’s disease, have been reported in patients with pathologically proven multiple system atrophy, but their causal contribution to the disease remains unclear.^77,78^ Genome-wide association studies in European and North American cohorts also failed to identify common single nucleotide polymorphisms significantly associated with multiple system atrophy.^58,79^ The identity of genetic factors causing or contributing to multiple system atrophy development therefore remains largely unknown and may differ between European and Asian natives.

Here we present a large and complex European pedigree including neuropathologically confirmed multiple system atrophy and Parkinson’s disease with dementia, as well as multiple cases of Parkinson’s disease and dementia in previous generations.

Neuropathological examination of the index case IV_22_ confirmed the diagnosis of multiple system atrophy of cerebellar type,^8,66^ with additional very early Alzheimer pathology,^45–48,80,81^ argyrophilic grain disease,^49^ microangiopathic changes and amyloid angiopathy of cerebral and leptomeningeal vessels.^50,51^

Neuropathological examination of her paternal cousin (family member IV_25_), who died with Parkinson’s disease with dementia, disclosed advanced diffuse Lewy body disease.^5,43,81^ In line with the clinical presentation at advanced disease of dementia with gaze palsy, frontal release signs, retrocollis and speech apraxia indicating a concomitant tau pathology, we also found middle-stage Alzheimer pathology,^45–48,80,81^ advanced argyrophilic grain disease,^49^ as well as cerebral and leptomeningeal microangiopathic changes in family member IV_25_.^51^

In both the index multiple system atrophy case and her paternal cousin with Parkinson’s disease with dementia, we observed astrogliosis and microglial activation as shown in previous reports.^82^ However, α-synuclein pathology displayed a different pattern of spreading and glial intracellular aggregation in these two cases. While microglial and astroglial α-synuclein inclusions were seen in the Parkinson’s disease with dementia case, this type of cellular pathology was not observed to accompany the GCIs in the multiple system atrophy case. Whether this finding reflects a difference in the properties of the α-synuclein strains,^83^ which in turn dictates the disease phenotype, or it is rather a consequence of the different glial properties and neuroinflammatory responses in the two cases remains to be addressed.

While the presence of one first-degree relative with Parkinson’s disease may simply reflect the chance of occurrence of Parkinson’s disease in the aging population, the remarkable clustering of α-synucleinopathies observed in the present pedigree suggests the presence of shared genetic factors. Our multiple system atrophy patient, her paternal cousin with Parkinson’s disease with dementia and another male member of generation III from the maternal arm, who suffered in life from Parkinson’s disease, showed no variants of interest, copy number variations or DNA repeat expansions in genes that may cause or increase the risk of multiple system atrophy, multiple system atrophy mimicries or other known forms of genetic parkinsonism. These results support the contention that variants in one or more genes that play a role in the disease pathogenesis in this family remain to be identified. Given the strongly positive family history over both the maternal and paternal line, it is tempting to speculate that the index multiple system atrophy case may have inherited pathogenic gene variants from both sides, in turn contributing to the clinical and pathological pleomorphism observed between her and her paternal cousin (IV_25_) affected by Parkinson’s disease with dementia. Interestingly, subject IV_25_, but not the index multiple system atrophy case or the other family member with Parkinson’s disease from generation III, was a heterozygous *ApoE* ɛ4 carrier. This may have favoured the development of clinically overt dementia with diffuse Lewy body, as well as Alzheimer’s pathology in this case.^84^

The assessment of the prodromal Parkinson’s disease risk did not reveal probable prodromal Parkinson’s disease among the family members without clinically evident parkinsonism of generation IV to VI. One sibling of the spouse of the index case, whose families were genealogically related (Fig. 2), reached the required LR for possible prodromal Parkinson’s disease. Future phenoconversion of this or other pedigree members to overt Parkinson’s disease or another α-synucleinopathy might guide further genetic analysis.

Our study has some limitations. First, and most importantly, it is possible that the disease is not due to shared genetic variants in all the affected members of the family, and one (or more) phenocopies might be present in this large pedigree. The occurrence of phenocopies is well-known in large pedigrees, particularly those with relatively common and aetiologically complex phenotypes, such as Parkinson’s disease,^85^ and challenges the search for novel disease-causing genes. Second, a genetic contribution from both parental arms is possible based on the family tree analysis (and provided the rationale for including both individual III_12_ and IV_25_ in the genetic analysis), but cannot unfortunately be proven, because both parents of the index case were deceased at the time the study began and there was no DNA available. For the same reason, we could not verify the neurological diagnoses in the family members of generation I to III, even though detailed information was collected and cross-checked among different living family members. Third, the prodromal Parkinson’s disease risk was calculated without including instrumental markers with high LR, such as dopaminergic imaging or polysomnography, and most of the family members without clinically evident parkinsonism were below 50 years of age, i.e. with a low pre-test probability of suffering from Parkinson’s disease (0.2%).^42^ This might have generally led us to underestimate the risk for prodromal Parkinson’s disease in the pedigree. It is also unproven that the same risk factors predicting prodromal Parkinson’s disease equally pinpoint people at risk of developing multiple system atrophy in the future.

As seen in other neurodegenerative disorders, the identification of defects in genes implicated in hereditary forms of the disease sheds light also on cellular cascades impaired in individuals affected by sporadic forms of the same disorder. The presence of both advanced α-synuclein and tau pathology in family member IV_25_ and, to a lesser extent, in the index multiple system atrophy case, who typically died after a much shorter disease duration than her paternal cousin, point altogether towards severe deficits in cellular protein clearance in both cases.

New insights from ongoing genetic initiatives in people with Parkinson’s disease and multiple system atrophy, as well as future clinical and molecular genetic studies of the present family have the potential to unravel the involvement of novel genes associated with the development of α-synuclein pathology. Ultimately, elucidating the molecular pathogenesis of α-synucleinopathies such as multiple system atrophy and Parkinson`s disease is of crucial importance for developing effective neuroprotective therapies.

## Supplementary Material

fcac175_Supplementary_Data

## Abbreviations

GCIs =: glial cytoplasmic inclusions
LR =: likelihood ratio
LR+ =: positive likelihood ratio
LR1 =: neutral likelihood ratio
LR- =: negative likelihood ratio
