# Supplementary material for: A multiplex pedigree with pathologically confirmed multiple system atrophy and Parkinson’s disease with dementia
Source: Brain Commun. 2022 Jul 4;4(4):fcac175. doi: 10.1093/braincomms/fcac175 (PMC9291376; doi:10.1093/braincomms/fcac175)
Supplement: fcac175_Supplementary_Data [file fcac175_supplementary_data.pdf]

Supplementary Table I Evaluation scheme for the assignment of positive, negative and neutral likelihood ratios (LR+, LR-, LR1) for each prodromal Parkinson's disease risk factor and distribution thereof (in brackets) within the examined family members without overt parkinsonism (n=26, upper panel). Age distribution of the examined family members (lower panel).

**Item**	**LR+**<br>**(No. of cases)**	**LR1**<br>**(No. of cases)**	**LR-**<br>**(No. of cases)**	**Notes**
Sex	Male<br>(15)	-	Female<br>(11)	
Regular pesticide exposure	Positive history<br>(1)	Non-informative history<br>(1)	Negative history<br>(24)	
Occupational solvent exposure	Positive history<br>(8)	Non-informative history<br>(1)	Negative history<br>(17)	
Consume caffeinated beverage	<3 coffee/week<br>(0)	1-7 coffee/week<br>*or*<br>non-informative history<br>(26)	>1 coffee/day<br>(0)	
Smoking status/history	Non-smoker<br>(18)	Former smoker<br>*or*<br>non-informative history<br>(4)	Active smoker<br>(4)	LR1 for former smokers was based on recent observations that ease to quit smoking may represent an early sign of Parkinson's disease.[1-3]
Family history of Parkinson's disease<br>(1st degree relative)	Positive<br>(8)	Non-informative<br>(3)	Negative<br>(15)	We applied LR+ also for the 1st degree relatives of the multiple system atrophy index case.

Type II diabetes mellitus	Positive history<br>(1)	Non-informative history<br>(0)	Negative history<br>(25)	
Physical activity	<1 hour/week<br>(0)	Non-informative history<br>(25)	>1 hour/week<br>(1)	
Screening for REM sleep behaviour disorder	Answered yes to RBD-1Q<br>*and* ≥2/5 RBD-I items<br>(0)	Answered no to RBD-1Q,<br>but yes to 1/5 RBD-I item<br>(1)	Answered no to RBD-1Q<br>*and* 5/5 RBD-I items<br>(25)	
Excessive daytime somnolence	MDS-UPDRS I item 8 ≥3<br>*and*<br>NMSS item 3+4 ≥8<br>*and*<br>answered yes to STOP-Bang item 2<br>(1)	Questionnaires indicate daytime somnolence below threshold for LR+<br>(10)	MDS-UPDRS I, NMSS and STOP Bang exclude daytime somnolence<br>(15)	
Olfactory loss	Score below threshold<br>(0)	Score ≤2 points above threshold<br>(8)	Score >2 points above threshold<br>(18)	Assessed with the 16-items Sniffin' Sticks (age- and sex-specific thresholds reported in Oleszkiewicz et al., 2019)[4]
Constipation	Treatment for constipation required more than once/week<br>*or*<br>spontaneous bowel movements less than every other day<br>(1)	Constipation tendency not reaching the defined thresholds for LR+ and LR-<br>(4)	SCOPA-AUT, NMSS, UMSARS and MDS-UPDRS I screen negative for constipation<br>(21)	

Urinary dysfunction	MDS-UPDRS item 10≥1<br>*or*<br>UMSARS item 10≥1<br>(2)	Non-informative history<br>(8)	MDS-UPDRS item 10=0<br>*and*<br>UMSARS item 10=0<br>(16)	Medical history excluded alternative causes (e.g. prostate hypertrophy, pelvic prolapse) for assignment of LR+.
Severe erectile dysfunction	Need of medication to engage in sexual activity<br>*or*<br>impossible sexual activity<br>(1)	Presence of sexual dysfunction, but not requiring medical intervention, non-informative history<br>*or*<br>female sex<br>(14)	SCOPA-AUT and UMSARS screened negative for sexual dysfunction<br>(11)	
Orthostatic hypotension	Documented orthostatic hypotension,[5] but no laboratory confirmation available or possible influence of medications[6]<br>(LR+ of 3.2)<br>(2)	No orthostatic test available<br>*or*<br>borderline findings<br>(3)	Orthostatic hypotension excluded on standing test[7]<br>(21)	
Depression ± anxiety	Positive history<br>*or*<br>use of antidepressants<br>*or*<br>MDS-UPDRS I item 3≥2<br>*or*<br>NMSS item 10≥6<br>(3)	Anxiety (MDS-UPDRS I item 4≥2), but no depression present[8]<br>(3)	No evidence of depression or anxiety at medical history, MDS-UPDRS or NMSS<br>(20)	

Global cognitive deficit	MoCA score ≥1.5 SD below mean	MoCA score between -1.5 SD and mean	MoCA score above mean	MoCA age-, sex- and education adjusted normative values reported under[9]
	(0)	(6)	(20)	
Subthreshold parkinsonism	UPDRS III ≥7	UPDRS III 3-6	UPDRS III <3	We excluded items on postural and action tremor.[8]
	(10)	(6)	(10)	Two movement disorder experts (PM, KS), who were blinded to the family tree, post-hoc rated the UPDRS-III score based on video-recordings of the motor examinations.

Age distribution in the examined family members without overt parkinsonism

**Age range**	**N**
< 50	12
50-54	2
55-59	2
60-64	1
65-59	3
70-74	3
75-79	2
> 80	1

LR = likelihood ratio; LR+ = test positive likelihood ratio; LR1 = neutral likelihood ratio; LR- = test negative likelihood ratio; RBD-1Q = RBD – 1 question; RBD-I = Innsbruck RBD Inventory; MDS-UPDRS = Movement Disorders Society – Unified Parkinson's disease rating scale; NMSS = Non-Motor Symptoms Scale; STOP Bang = Snoring, Tired, Observed (apnea), (high blood) Pressure, Body mass index, Age, Neck size, (male) Gender sleep apnea questionnaire; SCOPA-AUT = Scale for Outcomes in Parkinson's Disease – Autonomic Domains; UMSARS = Unified Multiple System Atrophy Rating Scale; MoCA = Montreal Cognitive Assessment; SD = standard deviation.

Supplementary Table 2 Detailed read depth distribution in the genes of interest in the WES of individuals III_{12}, IV_{22}, and IV_{25}.

Gene	Average read depth	Median read depth	Percentage of bases covered ≥30x	Percentage of bases covered ≥20x	Percentage of bases covered ≥10x	Individual
ABI3	116,9772727	104,5	87,59469697	94,12878788	100	III_{12}
ABI3	92,41856061	73	97,53787879	100	100	IV_{22}
ABI3	122,8584392	124	100	100	100	IV_{25}
ATN1	230,1029297	214	100	100	100	III_{12}
ATN1	198,2302307	200	99,79258491	100	100	IV_{22}
ATN1	113,1122306	115	99,72012315	100	100	IV_{25}
ATP13A2	191,9574021	183	100	100	100	III_{12}
ATP13A2	165,2500657	160	100	100	100	IV_{22}
ATP13A2	128,2801587	128	99,73544974	99,73544974	99,73544974	IV_{25}
ATXN1	260,8353576	239	100	100	100	III_{12}
ATXN1	233,9719973	218	100	100	100	IV_{22}
ATXN1	131,8006536	134	100	100	100	IV_{25}
ATXN2	167,3050933	135	98,15935451	99,92435703	100	III_{12}
ATXN2	145,6323752	112	97,22642461	98,89056984	100	IV_{22}
ATXN2	78,56444444	78	90,98039216	95,26797386	98,16993464	IV_{25}
ATXN3	142,4649254	146,5	100	100	100	III_{12}
ATXN3	128,0208955	130	99,10447761	100	100	IV_{22}
ATXN3	85,76724931	85	100	100	100	IV_{25}
ATXN7	195,1309042	183	100	100	100	III_{12}
ATXN7	171,2874494	155	100	100	100	IV_{22}
ATXN7	104,157133	106	94,34872502	97,03652653	99,58649207	IV_{25}
ATXN8OS	196,688588	201	100	100	100	III_{12}
ATXN8OS	178,9129594	175	100	100	100	IV_{22}
ATXN8OS	126,9074447	127	100	100	100	IV_{25}
C9orf72	131,1330495	114	98,93560404	99,30814263	100	III_{12}
C9orf72	121,7046301	118	98,66950506	99,8935604	100	IV_{22}
C9orf72	76,86009649	77	100	100	100	IV_{25}

CACNA1A	212,0569548	199	97,64586761	98,43057841	99,97468675	III_{12}
CACNA1A	180,1577016	169	97,20288571	98,56980129	99,91140362	IV_{22}
CACNA1A	100,3890271	99	98,4941731	99,69883462	100	IV_{25}
CHCHD2	144,53125	188	79,375	100	100	III_{12}
CHCHD2	110,81875	136,5	66,25	75	100	IV_{22}
CHCHD2	117,1913215	120	100	100	100	IV_{25}
COQ2	139,7481679	127	94,87008661	99,8667555	100	III_{12}
COQ2	121,4457029	117	93,33777482	95,53630913	99,73351099	IV_{22}
COQ2	67,41864139	63	93,12796209	100	100	IV_{25}
CYP27A1	259,486317	228	100	100	100	III_{12}
CYP27A1	237,8620296	213	99,37286203	100	100	IV_{22}
CYP27A1	90,44423559	89	96,80451128	100	100	IV_{25}
DCTN1	237,5143121	217,5	99,81532779	100	100	III_{12}
DCTN1	198,9275162	190	99,35364728	100	100	IV_{22}
DCTN1	99,5437776	99	100	100	100	IV_{25}
DNAJC6	233,4626401	212	100	100	100	III_{12}
DNAJC6	201,5395392	180,5	100	100	100	IV_{22}
DNAJC6	102,8510127	103	100	100	100	IV_{25}
EDN1	201,5837616	182	99,89722508	99,89722508	99,89722508	III_{12}
EDN1	192,2250771	167	100	100	100	IV_{22}
EDN1	83,90766823	83	100	100	100	IV_{25}
ELOVL7	117,04811	107	100	100	100	III_{12}
ELOVL7	114,4742268	111	98,39633448	100	100	IV_{22}
ELOVL7	84,40068886	85	100	100	100	IV_{25}
FBXO47	135,2498141	135	92,93680297	94,12639405	100	III_{12}
FBXO47	109,1836431	115	89,88847584	96,50557621	100	IV_{22}
FBXO47	85,98896247	84	100	100	100	IV_{25}
FBXO7	261,4629342	256	99,79263867	100	100	III_{12}
FBXO7	219,6215656	196	97,8745464	100	100	IV_{22}
FBXO7	94,59153176	95	100	100	100	IV_{25}

FMR1	85,69020716	67	91,90207156	96,42184557	99,81167608	III_{12}
FMR1	144,6723164	121	99,19962335	100	100	IV_{22}
FMR1	105,5687204	106	100	100	100	IV_{25}
FXN	115,2453271	92	95,21028037	100	100	III_{12}
FXN	106,203271	100	99,64953271	99,64953271	100	IV_{22}
FXN	82,02652106	71	93,44773791	99,06396256	100	IV_{25}
GBA	321,5225479	330	100	100	100	III_{12}
GBA	264,5806088	279	100	100	100	IV_{22}
GBA	133,4264432	140	100	100	100	IV_{25}
KLHL1	104,322104	94	93,20330969	99,46808511	100	III_{12}
KLHL1	95,98049645	74	97,69503546	100	100	IV_{22}
KLHL1	90,396	92	100	100	100	IV_{25}
LMNB1	197,8728407	200	98,80038388	100	100	III_{12}
LMNB1	166,9980806	166	93,23416507	96,54510557	98,80038388	IV_{22}
LMNB1	79,31629756	76	97,27427598	99,6592845	100	IV_{25}
LRP10	258,358209	258	100	100	100	III_{12}
LRP10	217,2224813	210	100	100	100	IV_{22}
LRP10	128,19972	131	100	100	100	IV_{25}
LRRK2	184,5601253	171	99,60830396	100	100	III_{12}
LRRK2	166,4338687	165	99,47773861	100	100	IV_{22}
LRRK2	76,03111814	75	99,76265823	100	100	IV_{25}
MAPT	164,2879929	159	98,93664156	100	100	III_{12}
MAPT	150,6437749	138	94,90474081	99,24678777	100	IV_{22}
MAPT	160,6080212	156	100	100	100	IV_{25}
NOP56	206,8442945	181	100	100	100	III_{12}
NOP56	165,7088252	141	97,58291175	99,94378865	100	IV_{22}
NOP56	99,61512605	104	100	100	100	IV_{25}
PARK7	154,8255495	147	100	100	100	III_{12}
PARK7	139,8173077	144	100	100	100	IV_{22}
PARK7	78,20877193	76	100	100	100	IV_{25}

PINK1	187,8941662	185	95,81827568	97,16055756	99,74186887	III_{12}
PINK1	177,7454827	179	95,04388229	100	100	IV_{22}
PINK1	94,12428408	97	89,11798396	91,52348225	94,1580756	IV_{25}
PLA2G6	241,7946429	238	100	100	100	III_{12}
PLA2G6	219,0128106	211	100	100	100	IV_{22}
PLA2G6	109,1460987	107	100	100	100	IV_{25}
PLCG2	261,5030948	253	100	100	100	III_{12}
PLCG2	230,0094083	218	100	100	100	IV_{22}
PLCG2	99,48367562	99	100	100	100	IV_{25}
POLG	241,6631471	226	100	100	100	III_{12}
POLG	215,348957	200	100	100	100	IV_{22}
POLG	109,1792228	111	100	100	100	IV_{25}
PPP2R2B	194,7810786	187,5	98,30895795	100	100	III_{12}
PPP2R2B	176,6599634	169	96,34369287	98,85740402	100	IV_{22}
PPP2R2B	101,3548193	96,5	100	100	100	IV_{25}
PRKN	204,9926108	207	100	100	100	III_{12}
PRKN	175,283867	172	100	100	100	IV_{22}
PRKN	101,9278056	97	100	100	100	IV_{25}
PRNP	141,4852136	114	100	100	100	III_{12}
PRNP	133,4556407	112	100	100	100	IV_{22}
PRNP	144,7139108	149	100	100	100	IV_{25}
PTRHD1	180,129902	157	100	100	100	III_{12}
PTRHD1	179,3112745	176,5	100	100	100	IV_{22}
PTRHD1	111,893617	115	100	100	100	IV_{25}
RAB39B	105,9213313	87	99,39485628	100	100	III_{12}
RAB39B	208,1951589	186	100	100	100	IV_{22}
RAB39B	167,9750779	173	100	100	100	IV_{25}
SHC2	161,372077	137	83,01237964	83,01237964	93,87895461	III_{12}
SHC2	140,8328748	127	83,01237964	83,01237964	93,87895461	IV_{22}
SHC2	103,0723722	117	78,80528432	80,47099368	80,7007467	IV_{25}

SNCA	193,1625767	186	100	100	100	III_{12}
SNCA	170,8282209	170	100	100	100	IV_{22}
SNCA	93,31603774	91	100	100	100	IV_{25}
SPG11	194,3629502	187	100	100	100	III_{12}
SPG11	166,7891949	165	99,62923729	99,84110169	100	IV_{22}
SPG11	91,72975184	91	100	100	100	IV_{25}
SPG7	265,7122153	260	97,51552795	99,96549344	100	III_{12}
SPG7	224,6017943	227,5	95,96273292	99,62042788	100	IV_{22}
SPG7	98,18307856	98	97,61820121	99,11126911	100	IV_{25}
SYNJ1	111,9231279	104	97,57013475	100	100	III_{12}
SYNJ1	95,21206097	84	94,56593771	96,86326486	100	IV_{22}
SYNJ1	79,68597248	78	100	100	100	IV_{25}
TBP	250,321875	208	100	100	100	III_{12}
TBP	201,9351563	175	100	100	100	IV_{22}
TBP	102,6088235	89	91,37254902	95,98039216	100	IV_{25}
TMEM230	163,7755102	182	93,46938776	100	100	III_{12}
TMEM230	125,5428571	157	86,93877551	86,93877551	98,7755102	IV_{22}
TMEM230	70,20403587	70	100	100	100	IV_{25}
VPS13C	94,94674984	80	85,66133261	93,06572643	99,13208571	III_{12}
VPS13C	85,3072959	72	82,09022692	91,98987433	98,11047826	IV_{22}
VPS13C	77,20255183	74	99,63671806	100	100	IV_{25}
VPS35	159,8833181	159	94,84958979	99,95442115	100	III_{12}
VPS35	132,6804923	126,5	92,84412033	94,34822242	95,80674567	IV_{22}
VPS35	86,78000836	88	100	100	100	IV_{25}

References

1. Ritz B, Lee PC, Lassen CF, Arah OA. Parkinson disease and smoking revisited: ease of quitting is an early sign of the disease. *Neurology*. Oct 14 2014;83(16):1396-402. doi:10.1212/wnl.0000000000000879

2. Moccia M, Erro R, Picillo M, et al. Quitting smoking: an early non-motor feature of Parkinson's disease? *Parkinsonism Relat Disord*. Mar 2015;21(3):216-20. doi:10.1016/j.parkreldis.2014.12.008

3. Mahlknecht P, Gasperi A, Djamshidian A, et al. Performance of the Movement Disorders Society criteria for prodromal Parkinson's disease: A population-based 10-year study. *Mov Disord*. Mar 2018;33(3):405-413. doi:10.1002/mds.27281

4. Oleszkiewicz A, Schriever VA, Croy I, Hähner A, Hummel T. Updated Sniffin' Sticks normative data based on an extended sample of 9139 subjects. *European archives of oto-rhino-laryngology : official journal of the European Federation of Oto-Rhino-Laryngological Societies (EUFOS) : affiliated with the German Society for Oto-Rhino-Laryngology - Head and Neck Surgery*. Mar 2019;276(3):719-728. doi:10.1007/s00405-018-5248-1

5. Freeman R, Wieling W, Axelrod FB, et al. Consensus statement on the definition of orthostatic hypotension, neurally mediated syncope and the postural tachycardia syndrome. *Clin Auton Res*. Apr 2011;21(2):69-72. doi:10.1007/s10286-011-0119-5

6. Fanciulli A, Leys F, Falup-Pecurariu C, Thijs R, Wenning GK. Management of Orthostatic Hypotension in Parkinson's Disease. *Journal of Parkinson's disease*. 2020;10(s1):S57-s64. doi:10.3233/jpd-202036

7. Fanciulli A, Campese N, Wenning GK. The Schellong test: detecting orthostatic blood pressure and heart rate changes in German-speaking countries. *Clin Auton Res*. Aug 2019;29(4):363-366. doi:10.1007/s10286-019-00619-7

8. Berg D, Postuma RB, Adler CH, et al. MDS research criteria for prodromal Parkinson's disease. *Mov Disord*. Oct 2015;30(12):1600-11. doi:10.1002/mds.26431

9. NACC. Means and SD for the UDS3 neuropsychological battery in cognitively normal participants. Accessed 02 February, 2022. https://files.alz.washington.edu/documentation/uds3-means.pdf
